# Expression of messenger molecules and receptors in rat and human sphenopalatine ganglion indicating therapeutic targets

**DOI:** 10.1186/s10194-016-0664-3

**Published:** 2016-09-01

**Authors:** Anna Steinberg, Simona D Frederiksen, Frank W Blixt, Karin Warfvinge, Lars Edvinsson

**Affiliations:** 1 Karolinska Institutet, Department of Clinical Neuroscience, Division of Neurology, Karolinska University Hospital Solna, 171 76 Stockholm, Sweden; 2 Department of Clinical Sciences, Division of Experimental Vascular Research, Lund University, Lund, Sweden; 3 Department of Clinical Experimental Research, Glostrup Hospital, University of Copenhagen, Glostrup, Denmark; 4 Department of Neurology, Karolinska University Hospital Solna, S-171 76 Stockholm, Sweden

**Keywords:** Sphenopalatine ganglion, Botox receptors, BoNT-A, Parasympathetic signaling transmitters, 5-HT receptor agonists

## Abstract

**Background:**

Migraine and Cluster Headache (CH) are two primary headaches with severe disease burden. The disease expression and the mechanisms involved are poorly known. In some attacks of migraine and in most attacks of CH, there is a release of vasoactive intestinal peptide (VIP) originating from parasympathetic cranial ganglia such as the sphenopalatine ganglion (SPG). Patients suffering from these diseases are often deprived of effective drugs. The aim of the study was to examine the localization of the botulinum toxin receptor element synaptic vesicle glycoprotein 2A (SV-2A) and the vesicular docking protein synaptosomal-associated protein 25 (SNAP25) in human and rat SPG. Additionally the expression of the neurotransmitters pituitary adenylate cyclase activating polypeptide (PACAP-38), nitric oxide synthase (nNOS), VIP and 5-hydroxttryptamine subtype receptors (5-HT_1B,1D,1F_) were examined.

**Methods:**

SPG from adult male rats and from humans, the later removed at autopsy, were prepared for immunohistochemistry using specific antibodies against neurotransmitters, 5-HT_1B,1D,1F_ receptors, and botulinum toxin receptor elements.

**Results:**

We found that the selected neurotransmitters and 5-HT receptors were expressed in rat and human SPG. In addition, we found SV2-A and SNAP25 expression in both rat and human SPG. We report that all three 5-HT receptors studied occur in neurons and satellite glial cells (SGCs) of the SPG. 5-HT_1B_ receptors were in addition found in the walls of intraganglionic blood vessels.

**Conclusions:**

Recent focus on the SPG has emphasized the role of parasympathetic mechanisms in the pathophysiology of mainly CH. The development of next generation’s drugs and treatment of cranial parasympathetic symptoms, mediated through the SPG, can be modulated by treatment with BoNT-A and 5-HT receptor agonists.

## Background

Migraine and Cluster Headache (CH) are two primary headaches with severe disease burden. In particular, CH is an extremely painful disorder characterized by periods (clusters) of recurrent, unilateral attacks of excruciating pain with a retro-orbital maximum and attacks lasting from 15–180 min [1]. CH usually appears between 20 and 40 years of age and during its active phase the attacks occur from once every second day to 8 times a day. Most patients show ipsilateral symptoms such as conjunctival injection, lacrimation, nasal congestion, rhinorrhoea and forehead/facial sweating, indicating an ipsilateral parasympathetic dysfunction which has been proven by co-release of the parasympathetic messenger molecule VIP [2].

The mechanisms involved in migraine and CH are considered to differ but also share some aspects [3]. Cranial autonomic symptoms (CAS), i.e. parasympathetic symptoms, occur in both migraine and CH patients [4–6], indicating involvement of the trigemino-autonomic reflex with increased parasympathetic outflow [5–7], mediated through the sphenopalatine ganglion (SPG) [8, 9]. Markedly raised levels of VIP and, in particular, calcitonin gene-related protein (CGRP) have been measured during spontaneous CH attacks. These findings were considered as evidence of involvement of the cranial parasympathetic nervous system [2]. In human SPG the parasympathetic signaling transmitters in neural cell bodies consist mainly of VIP, PACAP, acetylcholine (ACh) and NOS [10].

Onabotulinumtoxin A (BoNT-A), more commonly known by the trade name Botox®, comes from *Clostridium botulinum*. It works by blocking the release of the neurotransmitter ACh, which has been found in SPG [11], by cleaving SNAP25, a protein necessary for ACh release from vesicles in nerve endings [12]. However, it is unknown if SNAP25, and SV-2A, are expressed in the SPG. A previous study from our group has shown the presence of SV-2A and SNAP25 in rat trigeminal ganglion (TG) [11]. Incubation with BoNT-A was shown to reduce the inflammation response elicited by organ culture of the TG.

Triptans are 5-hydroxytryptamine (5-HT) receptor agonists with a high affinity for the 5-HT_1B/1D/1F_ receptors, which generally are effective for aborting headache attacks of both migraine and CH. The multiple mechanism of action for 5-HT_1B_/_1D_ receptors includes vasoconstriction, inhibition of the release of vasoactive neurotransmitters by trigeminal nerves as well as inhibition of nociceptive neurotransmission [13, 14]. 5-HT_1F_ receptors is characterized by lack of vasoconstrictive properties [15]. 5-HT_1B_,_1D_ receptors have been localized in the human TG [14–16]. Activation of those receptors seems to be one of the triptans modes of action. Early clinical studies showed effects in CH [2], thus triptans might have a direct effect on human SPG.

The aim of the present work was to examine if rat and human SPG contain the SV2-A and SNAP25 proteins and, by extension, if BoNT-A might have a mechanism of action in SPG. Secondly, we aimed at investigating the expression of 5-HT_1B,1D,1F_ receptors and the SPG neurotransmitters (PACAP-38, nNOS and VIP). This will provide novel and greater understanding of the action mechanisms in the SPG and could increase the possibility for future drug developments for CH.

## Methods

Wistar or Sprague–Dawley male rats (n _Wistar_ = 9, n _Spraque-Dawley_ = 10, weight = approx. 250 g) were euthanized by CO_2_ inhalation followed by decapitation. The SPG was carefully dissected out, close to the time of euthanasia, by initially making an incision over the zygomatic bone. The zygomatic bone was cut at both extremities and removed. The exposed deep masseter muscle was removed. The fifth cranial nerve trunk was revealed, carefully cut, and pulled posteriorly. The SPG, situated against the dorsal part of the maxillary bone, is thereby disclosed. The entire ganglion was carefully dissected and placed in 4 % paraformaldehyde for 2–4 h, followed by incubation overnight in Sörensen’s phosphate buffer (pH 7.2) containing 10 % and 25 % sucrose in turn. Thereafter, the tissue was embedded in Yazulla embedding medium (30 % egg albumin and 3 % gelatin in distilled water) and 10 μm cryosections were cut in a cryostat (Thermo Scientific Microm HM560). The sections were stored at −20 °C until use.

The human SPG was collected at autopsy, within 48 h post-mortem, from three patients. The patients were without disorders related to the central nervous system. The specimens were fixed in 4 % paraformaldehyde followed by sucrose-cryoprotection in 10 % sucrose Tyrode solution. The tissue samples were kept at −80 °C until embedding and cryo-sectioning. The study followed the guidelines of the European Communities Council (86/609/ECC) and was approved by the Committee of the Animal Research of University of Szeged (I-74-12/2012) and the Scientific Ethics Committee for Animal Research of the Protection of Animals Advisory Board (XI./352/2012). The rat study was approved by the Regional Ethical Committee on Animal Research, Malmö/Lund, Sweden. (M43-07).

### Hematoxylin-Eosin (HE)

Cryosections of rat and human SPG were stained using Hematoxylin (Htx) and Eosin dyes (Htx 4 min, Eosin 1 min). The staining was done in order to examine the morphology and condition of the tissue.

### Immunohistochemistry

Rat and human sections were washed in phosphate buffered saline (PBS) containing 0.25 % TritonX (PBS-T) once for 15 min followed by application of the primary antibody (Table 1) with incubation overnight at +4 °C in moisturized incubation chambers. The following day, the sections were washed twice in PBS-T for 15 min prior to incubation with secondary antibodies (Table 2) for 1 h in room temperature. Finally, the sections were washed 2×15 min and mounted with Vectashield mounting medium containing 4’,6-diamidino-2-phenylindole (DAPI) (Vector Laboratories, Burlingame CA, USA). Two out of three human samples were subjected to antigen retrieval by 30–60 min incubation at room temperature and at +75 °C in citrate buffer (10 mM sodium citrate, pH 6) prior to immunohistochemistry.

**Table 1:** Overview of the primary antibodies

Primary antibody					
Product	Product ID	Host	Dilution	Detects	Company
PACAP-38	T-4473	Rabbit	1:500	Human and rat PACAP-38	Peninsula Laboratories, LLC, San Carlos, CA, USA
nNOS	N2280	Mouse	1:2500	NOS derived from brain	Sigma Aldrich, St. Louis, MO, USA
VIP (M-19)	sc-7841	Goat	1:100	C-terminus of mouse VIP	Santa Cruz Biotechnology, Santa Cruz, CA, USA
SNAP-25	S9684	Rabbit	1:100	N-terminus of human SNAP-25	Sigma-Aldrich, St. Louis, MO, USA
SV-2A	ab32942	Rabbit	1:1000	Amino acids 1–100 of rat SV2A	Abcam, Cambridge, UK
5-HT_1B_	ab13896	Rabbit	1:100	Amino acids 8–26 and 263–278 of 5HT1B	Abcam, Cambridge, UK
5-HT_1D_	ab13895	Rabbit	1:100	Amino acids 1–18 and 251–267 of rat 5HT1D	Abcam, Cambridge, UK
5-HT_1F_	SP4043P	Rabbit	1:200	N-terminus extracellular domain of human 5HT1F	Acris Antibodies, San Diego, CA, USA

**Table 2:** Overview of the secondary antibodies

Secondary antibody				
Product	Host	Against	Dilution	Company
Alexa Flour 488	Donkey	Anti-goat	1:400	Invitrogen, CA, USA
Alexa Flour 594	Goat	Anti-mouse	1:200	Invitrogen, CA, USA
Alexa Flour 594	Donkey	Anti-rabbit	1:400	Jackson Immunoresearch Laboratories, Inc., West Grove, PA, USA
FITC	Donkey	Anti-mouse	1:100	Jackson Immunoresearch Laboratories, Inc., West Grove, PA, USA
FITC	Goat	Anti-rabbit	1:100	Cayman Chemical, Ann Arbor, MI, USA

Double stainings were exclusively performed in rat SPG. The protocol described above was repeated twice and done sequentially. Each staining was performed three times to ensure reproducibility. Omission of primary antibodies served as negative controls. The sections were examined in an epifluorescence microscope (Nikon 80i, Tokyo, Japan) equipped with a Nikon DS-2MV camera. Finally, images were processed using Adobe Photoshop CS3 (v0.0 Adobe Systems, Mountain View, CA).

## Results

### Hematoxylin Eosin staining

**Fig. 1:**
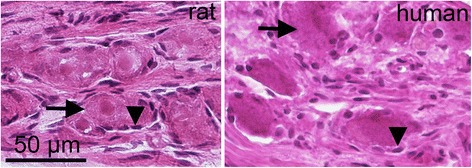
Hematoxylin-Eosin. The staining demonstrates neurons (*arrow*) and satellite glial cells (*arrow heads*) surrounding the neurons forming distinct units

HE staining of the SPG is shown in Fig. 1. The staining revealed neurons of different sizes, enveloped by a single layer of SGCs. These neuron/SGC units were intermingled between fibers. The morphology of the different rat SPGs was in general good, though minor tissue shrinkage was observed in some of the SPGs. Human SPG showed in general somewhat more shrinkage of the tissue.

### Rat SPG

#### Neurotransmitters

**Fig. 2:**
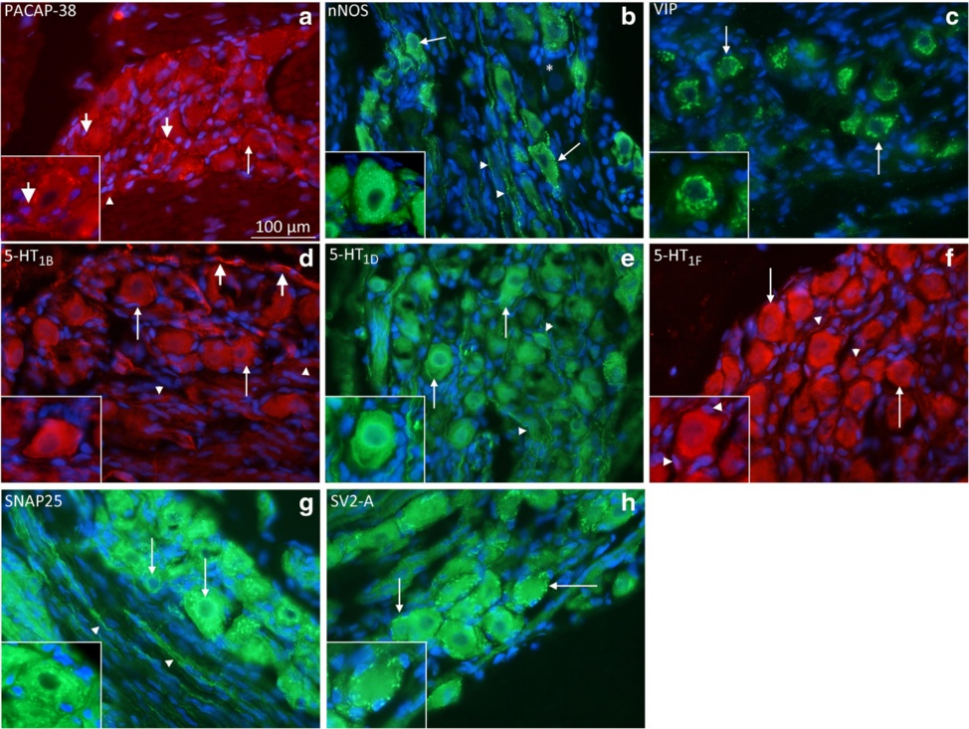
Rat SPG immunohistochemistry. **a** PACAP-38 immunohistochemistry. PACAP-38 was found in neurons (*thin arrows*), fibers (*arrow heads*), and very intense in the SGCs (*thick arrows*). **b** nNOS was expressed in the cytoplasm of many of the neurons (*thin arrows*), negative neurons were also found (*asterisk*). In addition, immunoreactive pearl-like fibers were observed (*arrow heads*). **c** VIP immunoreactivity was disclosed in a granular manner close to the neuronal nuclei, resembling endoplasmatic reticulum staining (arrows). **d** Serotonin receptor 5-HT_1B_ expression was found most neurons (*thin arrows*) and fibers (*arrow heads*), in addition to the vessel walls (*thick arrows*). **e** 5-HT_1D_ immunoreactivity was seen in many neurons (*arrows*) and nerve fibers (*arrow heads*). **f** 5-HT_1F_ immunoreactivity was seen in many neurons (*arrows*) and nerve fibers (*arrow heads*). **g** SNAP25 immunoreactivity was found in neurons (*thin arrows*) in the same granular pattern as described for VIP above. In addition, nerve fibers were immunoreactive (*arrow heads*). **h** SV2-A immunoreactivity was exclusively found in the SGCs (*arrows*)

PACAP-38 immunoreactivity was found in neurons and fibers, but individual differences were observed; not all animals displayed neuronal stainings. PACAP-38 immunoreactivity was found in the SGCs in all animals (Fig. 2a). nNOS was expressed in the cytoplasm of the neurons and in intraganglionic nerve fibers in a pearl-like manner around the neurons (Figs. 2b). In contrast to the homogeneous pattern of cytoplasmic stainings observed with PACAP-38 and nNOS immunohistochemistry, VIP immunoreactivity was found in a granular manner close to the neuronal nuclei, resembling endoplasmatic reticulum staining (Fig. 2c). By and large, VIP, PACAP-38 and nNOS were localized in the SPG neurons, while PACAP-38 alone was expressed in the SGCs.

#### 5-HT receptors

The 5-HT_1B_ receptor expression was found in numerous neurons and fibers, in addition to the vessel walls (Fig. 2d). 5-HT_1D_ and 5-HT_1F_ immunoreactivities were seen in many neurons and nerve fibers, but not in the vessel walls (Fig. 2e and f).

#### SNAP25 and SV2-A

SNAP25 immunoreactivity was found in most neurons in the same granular pattern as described for VIP above, but not in SGCs. In addition, nerve fibers were immunoreactive (Fig. 2g). SV2-A immunoreactivity was only found in the SGCs (Fig. 2h).

#### Double stainings

**Fig. 3:**
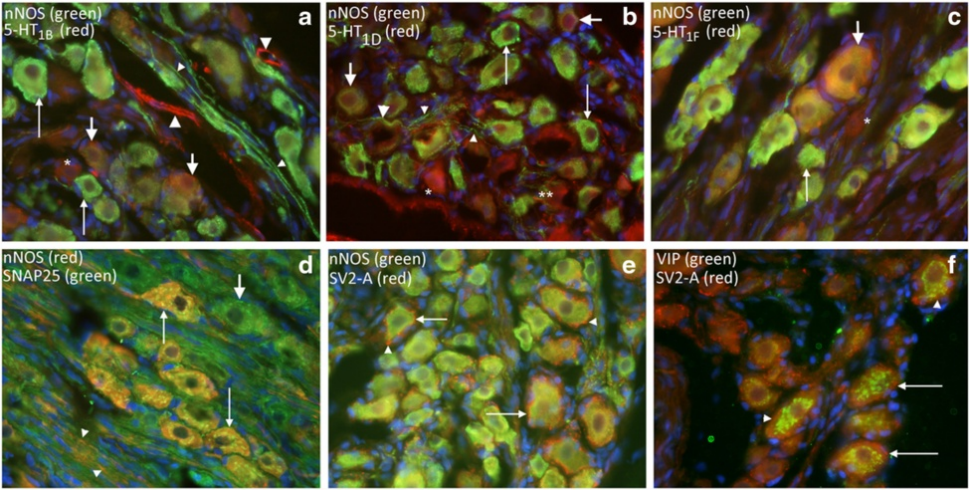
Rat SPG immunohistochemistry – double staining. **a** nNOS and 5-HT_1B_ double staining revealed neurons that expressed either nNOS (*thin arrows*) or 5-HT_1B_ (*asterisk*), but also both (*thick arrows*). Fibers were mostly nNOS positive (*thin arrow head*). Thick arrow heads point at 5-HT_1B_ positive vessels. **b** nNOS and 5-HT_1D_ double staining revealed neurons that expressed either nNOS (*thin arrows*) or 5-HT_1D_ (*asterisk*), but also both (*thick arrows*). The same pattern was found for the fibers; nNOS positive (*thin arrow head*), 5-HT_1D_ (*two asterisks*) or double stained (*thick arrow head*). **c** nNOS and 5-HT_1F_ double staining revealed neurons that expressed either nNOS (*thin arrows*) or 5-HT_1F_ (*asterisk*), but also both (*thick arrows*). **d** Double staining with nNOS and SNAP25 revealed that all nNOS immunoreactive neurons were also positive for SNAP25 (*thin arrows*). In addition, nNOS positive fibers were SNAP25 positive (*arrow heads*). SNAP25 immunoreactivity was found in most cells (*thick arrows*). **e** and **f** SV2-A (*arrow heads*) did not co-localize with either nNOS (*thin arrows*) or VIP (*thin arrows*). All images are shown with the same magnification

Co-localizations were found on one hand between nNOS and 5-HT_1B_ (Fig. 3a), 5-HT_1D_ (Fig. 3b), 5-HT_1F_ (Fig. 3c) and SNAP25 (Fig. 3d) on the other. SV2-A did not co-localize with either nNOS (Fig. 3e) or VIP in neurons (Fig. 3f).

**Fig. 4:**
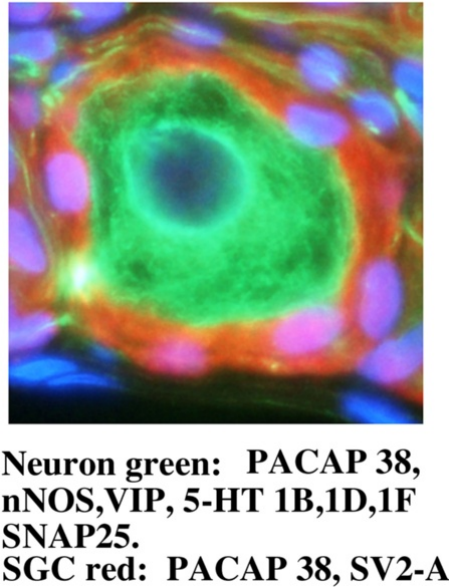
Overview over results found in rat SPG. The left side demonstrates antibody expression in the neurons and the right side in the SGCs. Only PACAP38 was found in both neurons and SGCs. SV2-A was only found in the SGCs

Summary of immunohistochemistry results are shown in Fig. 4.

### Human SPG

Individual differences were observed between the human samples in all stainings; two of the three specimens needed antigen retrieval to get the antibodies to recognize the antigen. In addition, many neurons in the human material contained intense autofluorescent lipofuscin in their cytoplasm

#### Neurotransmitters

**Fig. 5:**
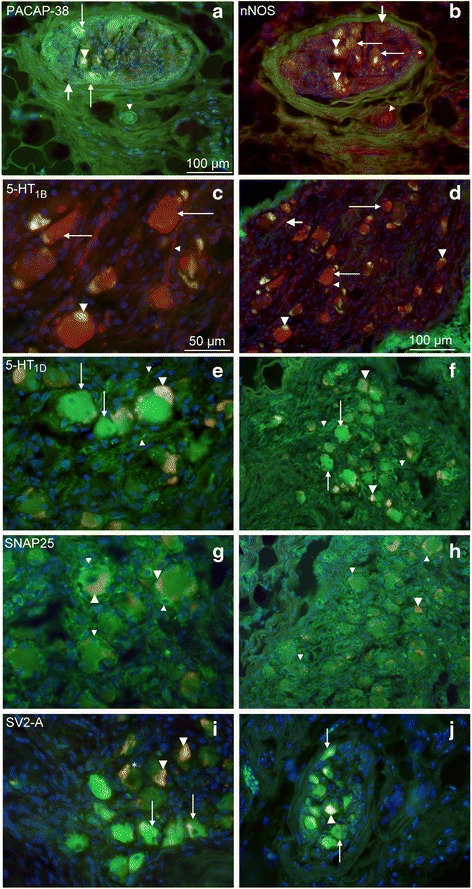
Human SPG immunohistochemistry. **a** PACAP-38 immunoreactivity was found in some neurons (*thin arrows*), in fibers (*thick arrow*) and vessel walls (*thin arrow head*). *Yellow* represent autofluorescent lipofuscin in the neurons (*thick arrow head*). **b** nNOS immunoreactivity was found in the neurons (*thin arrows*) and vessel walls (*thin arrow head*). *Thick arrow points* at a negative cell. *Yellow*; autofluorescent lipofuscin (*thick arrow head*). All images are in the same magnification. **c** and **d** 5-HT_1B_ immunoreactivity was found in most neurons (*thin arrows*), and in some fibers (*thin arrow heads*) and vessel walls (*thick arrow*). *Asterisk* indicates a negative cell. *Yellow*; autofluorescent lipofuscin (*thick arrow heads*). **e** and **f** 5-HT_1D_ immunoreactivity was seen in neurons (*thin arrows*) and fibers (*thin arrow heads*). Yellow; autofluorescent lipofuscin (*thick arrow head*) **g** and **h** SNAP25 immunoreactivity was exclusively found in SGCs (thin arrow heads). *Yellow*; autofluorescent lipofuscin (thick arrow head). **i** and **j** SV2-A immunoreactivity was confined to neurons (*thin arrows*). *Asterisk* indicates a negative cell. *Yellow*; autofluorescent lipofuscin (*thick arrow head*). The same magnifications are used throughout the panel (**c**-**j**)

PACAP-38 immunoreactivity was disclosed in some neurons, in nerve fibers and in vessel walls (Fig. 5a). nNOS immunoreactivity was found in neurons (Fig. 5b).

#### 5-HT receptors

5-HT_1B_ immunoreactivity was found in most neurons, in some fibers and in vessel walls (Fig. 5d and e). 5-HT_1D_ immunoreactivity was seen in neurons and fibers (Fig. 5f and g). 5-HT_1F_ immunoreactivity was not observed in the human material (using the available antibodies).

#### SNAP25 and SV2-A

SNAP25 immunoreactivity was exclusively observed in SGSs (Fig. 5h and i), while the SV2-A immunoreactivity was confined to neurons (Fig. 5j and k).

## Discussion

The present study is the first to examine the co-expression of signalling molecules and receptor elements in human and rat SPG. It is well known that triptans have clinically positive effects on acute pain in CH [17]. Thus, we asked the question if 5-HT_1B_, 5-HT_1D_ and 5-HT_1F_ receptors are expressed in neurons and SGCs in SPG. Importantly, we report that 5-HT_1B_, 5-HT_1D_ and 5-HT_1F_ receptors are expressed on most neurons in the rat SPG, which correlates well with the clinical effectiveness of triptans in CH. Here we demonstrate that all three 5-HT receptor subtypes occur in neurons and SGCs of the rat SPG. However, the 5-HT_1F_ receptor was only found in rodent material, possibly due to the antigenic properties of the used antibody. In addition 5-HT_1B_ receptors occur in the intraganglionic blood vessels, putatively indicating a possible vasomotor role. Previous studies have revealed expression of the parasympathetic signaling transmitters VIP, PACAP and nNOS in rat [18] and human [19] SPG. The results in the present study are in concert with these earlier studies. We found that both species contain SV2-A and SNAP25, elements involved in ACh neurotransmission, which has not been described earlier, however with a mixed expression. In rat, SNAP25 was expressed in neurons and fibers, but with SV2-A in the SGCs. In humans, SNAP25 was expressed in the SGCs, but SV2-A in the neurons. SNAP25 was mainly seen in the SGCs, while in man the neurons expressed Botox receptors elements SV2-A (opposite in rat). This could indicate that some effect of BoNT-A could occur in SPG provided it reaches this structure. The anatomical proximity of facial/temporal injection sites of BoNT-A in the PREEMPT protocol is much closer to the SPG than to the TG. The significance of the differential expression of SNAP25 and SV2-A is unclear but perhaps the localization of the receptor elements might suggest a potential target site of botulinum toxin if it has access to the receptor site.

Treatment with BoNT-A in adults with chronic migraine (CM) has shown safety and efficacy [20, 21]. Pilot studies of SPG injection of BoNT-A for treatment of CM as well as in chronic CH (CCH) have showed promising results [22, 23]. A previous study has shown presence of SV-2A and SNAP25 protein with same location in the TG [11]. The present results illustrate a possible site/mechanism of action for BoNT-A in CH. There is however no data available for an effect of BoNT-A in CH. The work provides anatomical rationale for this possibility, and given the proximity of the SPG to injection sites used in BoNT-A therapy it might be considered at least. Recent work has focused on neuromodulation of the SPG using e.g. specific SPG stimulation [24, 25].

An issue for the present work is if BoNT-A has a theoretical possibility to work as medical prophylaxis in CH. Earlier studies have shown varying results regarding BoNT-A as prophylactic treatment for CH. Twelve CCH patients were included in an open study where BoNT-A was given as an add-on therapy, i.e. prior prophylactic medication was continued [26]. BoNT-A was injected according to a standardised protocol, ipsilateral to the pain. Four of the twelve patients showed improvement [26]. A pilot study where BoNT-A was injected towards the SPG in CCH patients showed at least 50 % reduction of attack frequency in five out of ten patients [27]. So far, no randomised, placebo-controlled study regarding BoNT-A and treatment of CH has been performed.

Triptans were early found to abort CH attacks [28–30]. It was not until fairly recently that Ivanusic (2011) reported 5-HT_1D_ receptor immunoreactivity in nerve terminals around neurons in the rat SPG. These fibers were all CGRP positive and thus sensory in nature. They were traced back to the TG. Csati et al. (2012) showed CGRP positive fibers also in the human SPG, which agrees well with the present study.

Triptans are 5-HT_1B_/_1D_ receptor agonists with high affinity for 5-HT_1B_/_1D_ receptors, which are generally effective for aborting attacks in both migraine and CH. The multiple mechanism of action for 5-HT_1B_/_1D_ receptors includes vasoconstriction, inhibition of the release of vasoactive neuropeptides by trigeminal nerves as well as inhibition of nociceptive neurotransmission [13, 14]. Both 5-HT_1B/1D_ receptors have been localized in the human TG [14, 16]. Activation of those receptors seems to be one of the triptans modes of action. Triptans might have a direct effect on human SPG. So far, the issue has to be answered. We showed 5-HT_1B_ and 5-HT_1D_ immunoreactivity in SPG neurons, which suggests a role in modifying the activity in SPG. The variability in the 5-HT_1F_ expression is likely due to low specificity of these antibodies species. This issue is under current development.

Some limitations of our study need to be addressed. The human material is restricted to three SPG obtained at autopsy, due to difficulties to obtain those structures. Further, although the material has been carefully processed, we cannot exclude postmortem changes. In addition, our findings are purely anatomical and the question as to function may be addressed in subsequent work.

## Conclusion

Theoretically, our work provides anatomical indication, that both triptans and BoNT-A may have an effect on the SPG. Further randomised, placebo-controlled studies regarding especially BoNT-A treatment of CH are warranted. In addition this study also provides evidence for triptan effects in the SPG.
