# Erratum to: Expression of messenger molecules and receptors in rat and human sphenopalatine ganglion indicating therapeutic targets

**DOI:** 10.1186/s10194-016-0677-y

**Published:** 2016-09-20

**Authors:** Anna Steinberg, Simona D. Frederiksen, Frank W. Blixt, Karin Warfvinge, Lars Edvinsson

**Affiliations:** 1 Karolinska Institutet, Department of Clinical Neuroscience, Division of Neurology, Karolinska University Hospital Solna, 171 76 Stockholm, Sweden; 2 Department of Clinical Sciences, Division of Experimental Vascular Research, Lund University, Lund, Sweden; 3 Department of Clinical Experimental Research, Glostrup Hospital, University of Copenhagen, Glostrup, Denmark; 4 Department of Neurology, Karolinska University Hospital Solna, S-171 76 Stockholm, Sweden

Following the publication of the original article [1] it was brought to our attention that two minor errors were contained in the final article. Please see below for the sentences as they are now and how they should read upon revision:

**Abstract, Results:** We report that all three 5-HT receptors studied occur in neurons and satellite glial cells (SGCs) of the SPG.

This should read as:

We report that all three 5-HT receptors studied occur in neurons and nerve fibers of the SPG.

**Discussion, first paragraph, line 6:** Here we demonstrate that all three 5-HT receptor subtypes occur in neurons and SGCs of the rat SPG.

This should read as:

Here we demonstrate that all three 5-HT receptor subtypes occur in neurons and nerve fibers of the rat SPG.
